# IGFBP-2: a critical player in cancer and metabolism

**DOI:** 10.1186/1687-9856-2015-S1-O2

**Published:** 2015-04-28

**Authors:** George Werther, Steve Yau, Walid Azar, Matt Sabin, Vince Russo

**Affiliations:** 1 Murdoch Childrens Research Institute, Royal Children’s Hospital and Department of Paediatrics, University of Melbourne, Melbourne, VIC, Australia

## 

Insulin-like growth factor binding protein-2 (IGFBP-2) is widely abundant in fetal life and remains highly expressed in the brain throughout life. It plays a key role in targeting IGFs to their receptors in developing organs [1]. It is also highly expressed by a broad range of aggressive cancers such that its ablation reduces cancer growth. IGFBP-2 has also recently been recognised to play a key role in metabolic regulation.

We have been investigating the role and mechanisms of action of IGFBP-2, and have developed unique insights into its structure-function relationships. Our work has mostly focused on neuroblastoma cell lines of varying aggressiveness and motility, and our manipulations of the IGFBP-2 molecule have allowed functional characterisation.

We have demonstrated that the IGFBP-2 molecule contains a critical central basic region, the heparin-binding domain (HBD), which accounts for its binding to cell surface, critical in targeting IGFs to their receptors. Ablation of this domain dramatically reduces both proliferation and motility of these cancer cells, a process also involving integrin receptors [2].

This same region of the molecule also contains a Nuclear Localisation Sequence (NLS) accounting for the ability of IGFBP-2 to enter the cell nucleus. This process leads to induction of transcription of a range of cancer-promoting genes including VEGF, promoting angiogenesis in a chick embryo model, intrinsic to carcinogenesis. Ablation of the NLS blocks these processes [2,3].

Circulating IGFBP-2 is reduced in obesity and other insulin-resistant states and in animal models can enhance insulin sensitivity. It is regulated by the insulin sensitiser, leptin, and in a sheep model we have demonstrated that centrally administered leptin induces IGFBP-2 expression in skeletal muscle, via the sympathetic nervous system. Similarly, leptin applied directly to cultured skeletal muscle cells induces IGFBP-2 expression, leading to enhanced insulin sensitivity. This suggests that IGFBP-2 may mediate leptin’s insulin-sensitising effects [4].

IGFBP-2 also acts on visceral, but not subcutaneous adipose tissue, inhibiting fat accumulation, an effect not seen when the HBD region is mutated or integrin blockade is applied. Thus IGFBP-2 appears to play a beneficial role in regulating the function of visceral fat, which is critical in the pathogenesis of metabolic complications of obesity.

IGFBP-2 is thus a critical player in both cancer biology and in metabolic syndrome. There is potential for targeted pharmacologic manipulation of IGFBP-2 activity in order to reduce the morbidity and mortality of these major diseases in our society.
